# The hidden burden of viral meningitis in ethiopia: Etiology and clinical outcomes

**DOI:** 10.1371/journal.pone.0361291

**Published:** 2026-10-23

**Authors:** Derso Wale Mesele, Lema Ayele, Jemal Aman, Dawit Hailu Alemayehu, Ashenafi Alemu, Aschalew Gelaw, Yared Merid, Adane Mihret, Andargachew Mulu, Getachew Tesfaye Beyene

**Affiliations:** 1 Department of Medical Laboratory Science, College of Medicine and Health Science, Wolkite University, Wolkite, Ethiopia; 2 Armauer Hansen Research Institute, Addis Ababa, Ethiopia; 3 Department of Medical Laboratory Science, College of Health Science, Arsi University, Asella, Ethiopia; 4 Department of Medical Laboratory Science, College of Health Science, Madda Walabu University, Robe, Ethiopia; 5 Department of Medical Microbiology, School of Biomedical and Laboratory Sciences, College of Medicine and Health Sciences, University of Gondar, Gondar, Ethiopia; 6 College of Medicine and Health Sciences, Hawassa University, Hawassa, Ethiopia; Debre Markos University, ETHIOPIA

## Abstract

**Background:**

Viral meningitis remains a significant global public health problem, particularly in resource-limited settings where the diagnosis of viral etiology is mainly based on clinical assessment. This study aimed to identify the common viral etiologies of meningitis, describe their associated clinical features, and assess patient outcomes among individuals suspected of meningitis without evidence of bacterial meningitis.

**Methods:**

We conducted a multicenter study involving 430 patients clinically suspected of meningitis. Clinical data and cerebrospinal fluid samples (CSF) were collected following standard procedures. Viral nucleic acids were extracted from CSF specimens. Complementary DNA was prepared from viral RNA using reverse transcription. Viral nucleic acids were amplified and detected using qualitative conventional polymerase chain reactions. Patient outcomes were evaluated using the Glasgow Outcome Scale (GOS). Bivariate and multivariable logistic regression analyses were used to identify factors associated with unfavorable patient outcomes, with statistical significance set at a *p*-value < 0.05 at a 95% confidence level.

**Results:**

The prevalence of viral meningitis was 52.8% (227/430). Enteroviruses were the most common, 47.9% (206/430), followed by herpes simplex virus-1, 2.8% (12/430), herpes simplex virus-2, 1.4% (6/430), and Epstein-Barr virus, 0.7% (3/430). Overall, 6.7% (29/430) of study participants experienced unfavorable outcomes (GOS = 1–4). Factors independently associated with unfavorable outcomes included sex (AOR = 2.684, 95% CI = 1.177–6.119), duration from disease onset to hospital visit (AOR = 1.433, 95% CI = 1.043–1.967), and history of antibiotic intake before hospital visit (AOR = 2.784, 95% CI = 1.270–6.104).

**Conclusions:**

In this predominantly pediatric study, enteroviruses are the leading cause of viral meningitis in Ethiopia. Most study participants experienced favorable outcomes. Unfavorable outcomes were independently associated with sex, duration from disease onset to hospital visit, and history of antibiotic use before hospital visit. Further identification and characterization of Enteroviruses are warranted.

## Introduction

Viral meningitis remains a significant global public health problem caused by viral infection of the meninges [1,2]. A wide range of viruses can cause meningitis, with enteroviruses (EVs) being the predominant causative agents worldwide and disproportionately affecting young children [3,4]. Herpes viruses (herpes simplex virus type 1 (HSV-1), herpes simplex virus type 2 (HSV-2), varicella-zoster virus (VZV), Epstein-Barr virus (EBV)) and mumps virus are also common pathogens [2,3].

The clinical manifestations of viral meningitis are usually similar for the different causative agents, mainly characterized by fever, headache, neck stiffness, altered level of consciousness, nausea, vomiting, and photophobia, which makes it difficult to diagnose the disease at early stages [3,5]. In children, these signs and symptoms are usually atypical, which further increases the risk of delay and misdiagnosis. As a result, empirical antimicrobial treatment is a common practice in this setting despite a high percentage of causative viral agents, which have no proven benefit for them [5]. In such a situation, laboratory confirmation is essential.

Cerebrospinal fluid (CSF) analysis, particularly polymerase chain reaction (PCR), which detects viral DNA or RNA, is the gold standard for the diagnosis of viral meningitis. In recent years, advances in PCR have significantly improved detection [2,6]. Despite these advances, challenges remain in resource-limited settings like Ethiopia.

Even though viral meningitis is usually self‑limiting, it can be severe and result in unfavorable outcomes, especially in extreme ages and immunocompromised patients [7]. The prognosis may also worsen with a delayed diagnosis. Timely diagnosis can help to reduce hospitalization rates, length of hospital stays, healthcare costs, and adverse effects of unnecessary antibiotics [5,8]. Certain factors, including age, sex, comorbidity, delay in hospital presentation, antibiotic intake before hospital visit, and infection with a certain herpes virus, have been associated with unfavorable outcomes [9].

In Ethiopia, the viral etiology of meningitis is rarely identified due to limited infrastructure and skilled personnel [4,5]. Existing studies are scarce and often limited by small sample sizes, and most were from repository samples, which may not accurately reflect the clinical reality of viral meningitis or could potentially bias the results. More importantly, routine viral meningitis diagnosis is clinical. Therefore, there is a critical need for a large, multicenter molecular epidemiology study to integrate molecular diagnostic techniques with routine methods for etiologic investigation to improve the clinical management of suspected meningitis patients.

Thus, this study aimed to detect the etiology, clinical features, and outcomes of viral meningitis in Ethiopia. To the best of our knowledge, this study is the largest multicenter and most comprehensive study of viral meningitis in Ethiopia.

## Materials and methods

### Study setting

The study was conducted from 01/08/2023–30/06/2024 at five tertiary-level hospitals in Ethiopia: University of Gondar Comprehensive Specialized Hospital (UoGCSH), Tikur Anbessa Specialized Hospital (TASH), Yekatit 12 Hospital Medical College (YE12HMC), All Africa Leprosy Rehabilitation and Training (ALERT) Comprehensive Specialized Hospital, and Hawassa University Comprehensive Specialized Hospital (HUCSH). These study sites are geographically distributed across Ethiopia to ensure broad national representation. Tikur Anbessa Specialized Hospital, ALERT Comprehensive Specialized Hospital, and YE12HMC are located in Addis Ababa, in central Ethiopia. University of Gondar Comprehensive Specialized Hospital is located in Gondar, in northwest Ethiopia. Hawassa University Comprehensive Specialized Hospital is located in Hawassa, in southern Ethiopia.

### Inclusion criteria

Four hundred thirty meningitis-suspected patients who were PCR- and culture-negative for bacterial meningitis were included in this study.

### Specimen collection

Cerebrospinal fluid samples were collected aseptically from the suspected patient. Depending on the age of the patient, lumbar punctures were done using a needle measuring 1.5 inches (3.8 cm) to 3.5 inches (8.9 cm). The CSF sample was collected using sterile plastic tubes (Sartstedt, Germany) by following the standard operating procedure (SOP) and transported to the laboratory within 30 minutes of collection. Approximately 0.5 mL of CSF was transferred to a Nunc® CryoTube inside a Biosafety Level 2 cabinet and stored at −80 °C until used for further experiments.

### Nucleic acid extraction and PCR

Extraction of viral nucleic acid from CSF samples was performed according to the protocol provided with the High Pure viral nucleic acid Preparation Kit (Roche Life Sciences, Mannheim, Germany). The concentration and purity of DNA or RNA of the extract were determined using a Nanodrop Spectrophotometer (ND-1000; Nanodrop Technologies, Wilmington, DE) at 260, 260/280, and 260/230 nm.

For amplifying the viral DNA target, Platinum Taq Hot Start PCR Master Mix (Thermo Fisher Scientific) was used. The final volume of each PCR reaction was 25 µL of [2.5 µL of 10X buffer, 1.2 µL of 50 mM MgCl_2_, 1 µL of dNTP master mix, 1 µL of each forward and reverse primer, 0.2 µL of Taq polymerase, 5 µL of target DNA, and 13.1 µL of nuclease-free water]. Conventional PCR was performed using the Bio-Rad T100 thermal cycler. Amplification involved an initial denaturation at 94 °C for 3 min, followed by 35 cycles of denaturation at 94 °C for 10 s, annealing at 60 °C for 30 s, and extension at 72 °C for 30 s, with a final extension step at 72 °C for 5 min. The PCR products were visualized using a 2.5% agarose gel with ethidium bromide under UV light in a CS-DARKROOM, Cleaver Scientific.

For the RNA virus, a two-step reverse transcription polymerase chain reaction (RT-PCR) was conducted for the amplification of EVs and mumps. Enterovirus- and mumps-specific primers were used to detect all EVs and mumps [Table 1]. Reverse transcription was performed in a 20 µL reaction composed of 0.2 µL of random hexamer, 1 µL of 10 mM dNTP, 1.8 µL ultra-pure water treated with 0.1% diethylpyrocarbonate (DEPC), 10 µL template RNA, 1 µL of 100 mM DTT, 1 µL of RNase inhibitor (40 units/µL), 0.8 µL of 10 × RT buffer, and 5 µL of Superscript IV. The reaction mixture was then incubated at 65 ^°^C for 5 minutes to synthesize cDNA, and the reaction was inactivated by incubating at 55 ^°^C for 50 minutes and 80 ^°^C for 10 minutes. Polymerase Chain Reaction amplification was done by adding 5 µL cDNA to 20 µL PCR master mix (consisting of 5 µL of 5 Green Go Taq Flexi Buffer, 2.5 µL 25 mM MgCl_2_, 0.5 µL 10 mM dNTP, 0.3 µL of forward and reverse primers each (10 µM), 0.1 µL Go-Taq G2 Flexi DNA polymerase, and 10.3 µL molecular grade water). The thermal cycling was adjusted to 94 ^°^C for 2 minutes, and 35 cycles of denaturation at 94 ^°^C for 30 seconds, followed by annealing at 58 ^°^C for 30 seconds and extension at 72 ^°^C for 30 seconds. The amplicon was visualized on a 2.5% agarose gel. This protocol was obtained from the Go-Taq G2 Flexi DNA polymerase protocol.

**Table 1:** Primer sequences with their respective nucleotide length and target gene.

Virus	Gene	Primer	Sequence (5’ - 3’)	Size (bp)	Reference
Herpes simplex virus 1	*gpB*	HSV1-gpB-F	CCACCGTCAGCACCTTCAT	127	[10,11]
		HSV1-gpB-R	CGCTGGACCTCCGTGTAGTC		
Herpes simplex virus 2	gpD	HSV-2-gpD-F	CACCACTTAAATCCTAAGGTTCC	124	[12]
		HSV-2-gpD-R	CTGATGACATAATTGAGATTGCACCC		
Epstein-Barr virus	EBNA	EBV3-9F	CTGACACTTTAGAGCTCTGGAG	228	[10]
		EBV3-9R	GGCCCTGACCTTTGGTGAAGTCA		
Varicella-Zoster virus	DNAPol	VZV-DNApolF	GCGCTCTAACGTTCGAGAAAGT	60	[11,13]
		VZV-DNApolR	CGCATAGCCAACCAGTCTTTT		
Enteroviruses	5′ NTR	PanEV2–5′NTR F	CATGGTGCGAAGAGTCGATTG A	144	[5]
		PanEV2–5′NTR R	CACCCAAAGTAGTC GGTTCCGC		
Mumps virus	V/P	MumV-F	TTCAGGGAACCAACTCGTTGA	171	[10]
		MumV-R	CTTCGGAGGATGAGACCATGAT		

### Quality control

NATEVPOS-6MC for enterovirus, NATEBV-005 for Epstein-Barr Virus, and NATVZV-0005 for Varicella-Zoster virus from NATrol (Helvetica Health Care, Geneva, Switzerland) were used as viral positive controls, and RNase/DNase-free water was used as a negative test control to ensure the quality of the laboratory result.

### Data management and statistical analysis

The data were first coded, cleaned, and entered into Epi Info v 3.1, then transformed and analyzed with the Statistical Package for Social Sciences (SPSS) version 26 statistical software. The results of the analysis were presented in text, figures, or tables where appropriate. Categorical variables were summarized using frequencies and percentages. Skewed data were presented as the median with an interquartile range. The association between variables was assessed using binary logistic regression and multivariable regression analysis. All independent variables with *p* < 0.25 in bivariate analysis were included in multivariable analysis. The best-fit model was identified using forward logistic regression analysis. In all cases, *p* < 0.05 was considered statistically significant.

### Operational definitions

A suspected meningitis case was defined as a patient with a sudden onset of fever, neck stiffness, or other meningeal signs. A Confirmed viral meningitis case was any suspected case with viral nucleic acid detected by PCR or RT-PCR in the CSF [14,15].

The GOS was categorized as (1- death; 2- vegetative state; 3- severe disability; 4- moderate disability; 5- good recovery) at discharge. Later, the scores were dichotomized into a favorable outcome (GOS = 5) and an unfavorable outcome (GOS = 1–4) [9]. To ensure that all assessors had a common understanding of the scale criteria and to reduce interpersonal variability, we conducted a 6-hour orientation. Any scoring discrepancies were addressed through consensus discussions among the assessors, as described in our previous bacterial meningitis study.

### Ethical consideration

The study was performed in compliance with the Declaration of Helsinki and approved by the AHRI/ALERT Ethics Review Committee (AAERC) under protocol number PO-55–22. Data were collected after obtaining written informed consent from the study adult subjects or assent from parents/guardians for children or the unconscious.

## Results

### Demographic characteristics of the study participants

Across the five sites, a total of 430 patients with suspected meningitis and PCR negative for bacterial meningitis were included in this study. The age of participants ranged from 1 month to 81 years. Of these, 223 (51.9%) were male, indicating a nearly equal sex distribution. The majority of the study participants, 389 (90.5%), were children under 18 years of age. UoGCSH contributed the largest proportion of participants, with 141 (32.6%), followed by Y12HMC, which had 110 (25.6%) (Table 2).

**Table 2:** Sociodemographic characteristics of the study participants.

		UoGCS, n (%)	TASH, n (%)	Y12HMC, n (%)	ALERT-Hospital, n (%)	HUCSH, n (%)	Total (%)
Number of participants		141 (32.6)	36 (8.4)	110 (25.6)	106 (24.7)	37 (8.6)	430
Sex	Male	77 (54.6)	19 (52.8)	53 (48.2)	59 (55.7)	15 (40.5)	223 (51.9)
	Female	64 (45.4)	17 (47.2)	57 (51.8)	47 (44.3)	22 (59.5)	207 (48.1)
Age	≤5	83 (58.9)	27 (75)	87 (79.1)	89 (84)	32 (86.5)	318 (74)
	6–18	42 (29.8)	5 (13.9)	9 (8.2)	12 (11.3)	3 (8.1)	71 (16.5)
	>18	16 (11.3)	4 (11.1)	14 (12.7)	5 (4.7)	2 (5.4)	41 (9.5)

### Prevalence and pathogen distribution

Viral nucleic acid was detected in 227 (52.8%) participants. The leading viral agent was EVs 206 (47.9%), followed by HSV-1, 12 (2.8%), and HSV-2, 6 (1.4%). EBV was rare in this study, detected only in 3 (0.7%) participants. Although all participants were tested for VZV and the mumps virus, no cases were identified for either of the pathogens (Fig. 1).

**Fig 1:**
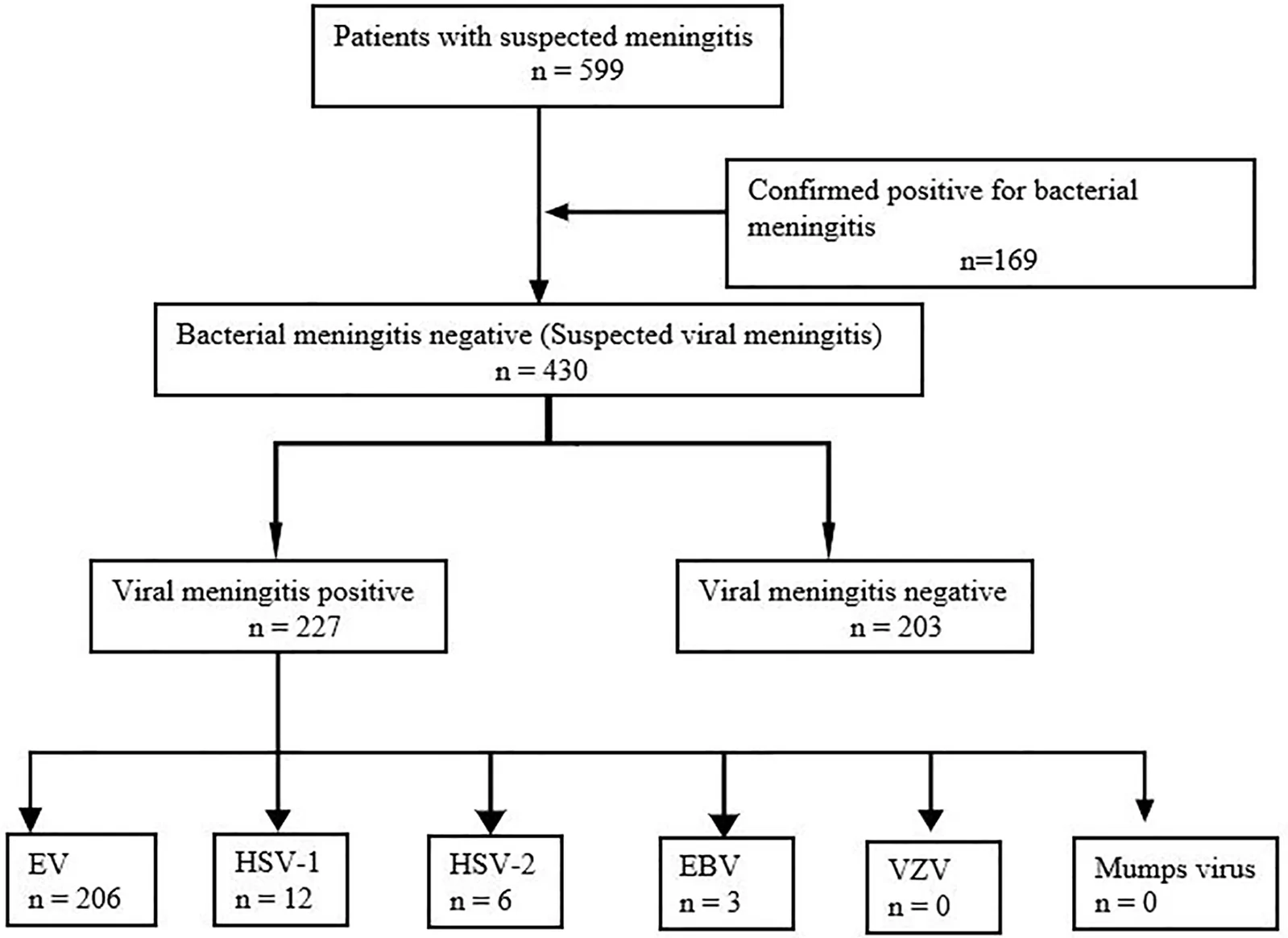
Inclusion of study participants.

As shown in Fig 1, the overall prevalence of viral meningitis among suspected cases, including those with confirmed bacterial infections, was 28.21% (169/599), 37.9% (227/599), and EV remained the most common pathogen irrespective of the study sites (Table 3) (Fig 2).

**Table 3:** Distribution of viral etiologies and study sites.

Study Site	Viral Etiology					Negative n(%)
	EV	HSV-1	HSV-2	EBV	Total positive	
UoGCS	53	5	0	1	59	82
TASH	26	0	0	0	26	10
Y12HMC	73	2	3	1	79	31
ALERT-Hospital	43	3	2	1	49	57
HU-Hospital	11	2	1	0	14	23
Total n (%)	206 (47.9%)	12 (2.8%)	6 (1.4%)	3 (0.7%)	227 (52.8%)	203 (47.2%)

**Fig 2:**
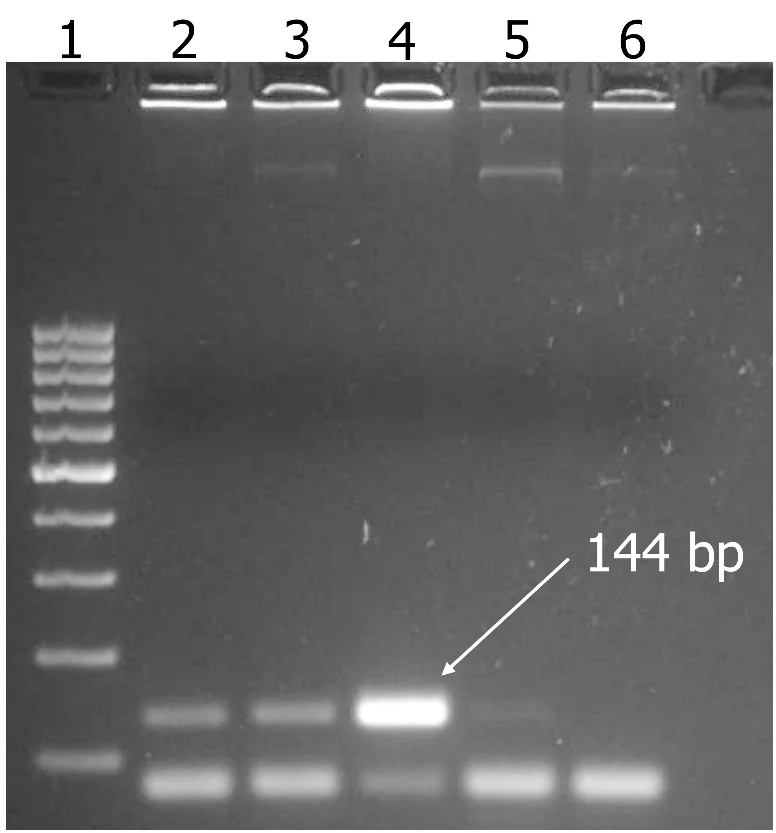
Agarose gel electrophoresis image of PCR amplification product bands of enterovirus by PanEV2-5′NTR on a 2.5% agarose gel. Lane 1 = 100-bp DNA ladder; Lane 2 = positive control; Lanes 3 and 4 = enterovirus-positive sample products 144 bp; and Lane 6 = NC, negative control.

### Association of clinical presentation with the virus detected

The common clinical presentations in this group of patients were fever 63% (271/430), vomiting 60.2% (259/430), lowered consciousness 43.7% (188/430), and neck stiffness 37.4% (161/430). These clinical presentations were more commonly seen among patients with PCR-confirmed viral meningitis compared to those who tested negative (Table 4).

**Table 4:** Association of clinical presentation with the viruses detected.

Clinical presentation	Virus detected					Not detected n = 203(%)	Total n = 430 (%)
	Total n = 227 (%)	EV n = 206 (%)	HSV-1 n = 12 (%)	HSV-2 n = 6 (%)	EBV n = 3 (%)		
Fever	161 (70.9)	150(72.8)	6 (50)	4 (66.7)	1 (33.3)	110(54.2)	271 (63)
Neck stiffness	126 (55.5)	113 (54.9)	6(50)	5 (83.3)	2 (66.7)	35(17.4)	161 (37.4)
Lowered consciousness	115 (50.7)	102 (49.5)	8 (66.7)	3 (50)	2 (66.7)	73 (36)	188 (43.7)
Seizure	77 (33.9)	63 (30.6)	11 (91.7)	3 (50)	0	53 (26.1)	130 (30.2)
Skin rash	6 (2.6)	6 (2.9%)	0	0	0	11 (5.4)	17 (4)
Vomiting	140 (61.7%)	133 (64.6%)	5 (41.7)	1 (16.7)	1 (33.3)	119 (58.6)	259 (60.2)

Among PCR-positive patients, the most common symptoms were fever 70.9% (161/227), followed by vomiting 61.7% (140/227), neck stiffness 55.5% (126/227), lowered consciousness 50.7% (115/227), and seizure 33.9% (77/227) (Table 4).

Among patients positive for EVs, fever was the most common sign and symptom observed in 72.8% (150/206) of the patients, followed by vomiting and neck stiffness, with rates of 64.6% (133/206) and 54.9% (113/206), respectively. Likewise, among HSV-1 positive patients, seizure was the most common sign and symptom observed in 91.7% (11/12) of the patients, followed by lowered consciousness in 66.7% (8/12), and 50% (6/12) for both neck stiffness and fever (Table 4).

### Outcomes and associated factors

The outcome of the 430 patients was assessed using GOS. The overall unfavorable outcome was observed in 29 (6.7%) patients. There was one death (0.2%), which occurred in an EV-positive patient. The outcome of all HSV-2-positive patients was favorable. Overall, 130/430 (30.2%) of participants had taken an antibiotic before a hospital visit (data in S1 Text).

In bivariate logistic regression analysis, sex, duration from disease onset to hospital visit, and history of antibiotic intake before hospital visit were found to be associated with unfavorable outcomes. These variables remained significantly associated with unfavorable outcomes in multivariate analysis: sex (AOR = 2.684, 95% CI = 1.177–6.119), duration from disease onset to hospital visit (AOR = 1.433, 95% CI = 1.043–1.967), and history of antibiotic intake before hospital visit (AOR = 2.784, 95% CI = 1.270–6.104). In this study, being positive for a viral pathogen did not have a significant association with unfavorable outcomes (Table 5).

**Table 5:** Bivariate and multivariable analysis of factors associated with unfavourable outcome (GOS = 1 to 4) at discharge in patients suspected of meningitis.

Variable		Outcome		Bivariate analysis		Multivariable analysis	
		Favorable	Unfavorable	COR (95% CI)	*P*-value	AOR (95% CI)	*P*-value
Age (Yrs.), median (IQR)		1(0.3-6)	0.8(0.3-5.45)	0.989(0.965-1.013)	0.369		
Sex	Male	214	9	1	0.024*	1	0.019*
	Female	187	20	2.543(1.130-5.721)		2.684(1.177-6.119)	
Fever	No	149	10	1	0.773		
	Yes	252	19	1.123(0.509-2.480)			
Neck stiffness	No	252	17	1	0.650		
	Yes	149	12	1.194(0.555-2.569)			
Lowered consciousness	No	228	14	1	0.370		
	Yes	173	15	1.412(0.664-3.003)			
Seizure	No	278	22	1	0.461		
	Yes	123	7	0.719(0.299-1.728)			
Skin rash	No	385	28	1	0.885		
	Yes	16	1	0.859(0.110-6.718)			
Vomiting	No	158	13	1	0.565		
	Yes	243	16	0.800(0.375-1.709)			
Duration of disease onset to hospital visit in days, mean±SD^a^		2.63 ± 1.30	3.28 ± 1.71	1.370(1.005-1.867)	0.046*	1.433(1.043-1.967)	0.026*
History of antibiotic intake before hospital visit	No	285	15	1	0.032*	1	0.011*
	Yes	116	14	2.293(1.073-4.902)		2.784(1.270-6.104)	
Virus detected	No	193	10	1	0.160		
	Yes	208	19	1.763(0.800-3.886)			

AOR adjusted odds ratio, CI confidence interval, COR crude odds ratio, IQR interquartile range, SD standard deviation, * Statistically significant, ^a^ Odds ratio decreases with a unit increase in the predictor variable

## Discussion

In this multicenter study, we investigated the etiologies, clinical features, and factors associated with unfavorable outcomes of viral meningitis.

Viral meningitis was confirmed in 227 (52.8%) participants. This finding is consistent with data from Southern Africa [16] and Iran [17], but findings were lower than those of the previous studies from Brazil [18], Korea [19], and Qatar [7]. To our knowledge, this reported prevalence is the highest in Ethiopia [4,5,20]. This may be due to the larger sample size and multicenter design of the current study, which enhanced population representativeness and case ascertainment across multiple settings, as well as comprehensive screening for common viral etiologies, which identified cases that had been missed by previous studies with narrower diagnostic panels.

Our findings identified EVs as the most common cause, accounting for 206 (47.9%) of viral meningitis cases. This aligns with the well-established understanding that EVs are the leading cause of viral meningitis by far [7,14,18]. Furthermore, our result aligns with the findings of earlier reports from Iran [21] and France [22]. The prevalence of EVs observed in this study is higher than in the previous studies in the country [4,5,20]. This could be due to the method of nucleic acid purification. In this study, nucleic acid extraction was done using a commercial kit to attain the maximum sensitivity (High Pure Viral Nucleic Acid Kit-Roche, Mannheim, Germany).

Contrary to studies from the Republic of Korea [19], Tunisia [23], and Australia [24], HSV-1 was a more common cause of viral meningitis in our study, with 2.8% (12/430), than HSV-2, 1.4% (6/430). This finding was in agreement with studies in Iran [25] and a prior Ethiopian study [20], possibly reflecting higher regional HSV-1 prevalence. EBV was rare, 0.7% (3/430), consistent with the low prevalence reported in Australia [24]. No cases of varicella-zoster virus or mumps virus were identified in this study. This is in agreement with the study in Ghana [26] and Qatar [7], which also report zero prevalence of mumps virus, possibly due to the effective vaccine, while the low detection of VZV may be related to the predominantly immunocompetent study population. These findings highlight the importance of considering local epidemiological contexts when interpreting viral meningitis etiologies.

In Ethiopia, clinicians routinely diagnose viral and bacterial meningitis based on clinical evaluation. However, differentiating between the two using clinical criteria alone remains highly challenging due to their significantly overlapping symptoms. In our study, fever 63% (271/430), vomiting 60.2% (259/430), lowered consciousness 43.7% (188/430), and neck stiffness 37.4% (161/430) were the most common presentations at admission. This is in agreement with the studies in Ethiopia [5], Denmark [9], China [27], Saudi Arabia [28], and Qatar [7].

Although viral meningitis is usually benign and self-limiting, it can result in unfavorable outcomes [29]. We found that 6.7% (29/430) of participants suspected of viral meningitis had unfavorable outcomes (GOS = 1–4) at discharge. Thus, 93.3% had a favorable outcome, with only one death, and the rest were discharged. This can be explained by the fact that most of our patients were young and immunocompetent.

Multivariate logistic regression analysis identified being female (AOR = 2.684, 95% CI = 1.177–6.119) as an independent factor associated with unfavorable outcomes. This indicates that females have about 2.7 times higher odds of experiencing an unfavorable outcome compared to males, aligning with findings from Denmark [9,30]. The reason for the sex differences is largely unknown, but may be due to females having a longer time to resolution of clinical manifestations and stronger immune responses to some pathogens [5,9].

Similarly, delayed presentation to hospital from disease onset (AOR = 1.433, 95% CI = (1.043–1.967) was independently associated with unfavorable outcomes, meaning a one-day delay increased the likelihood of an unfavorable outcome by 1.4 times. Poor health-seeking behavior and limited access may have contributed to these delays and have been highlighted previously as a risk factor for unfavorable outcomes in bacterial meningitis [31].

Approximately 27% (116/430) of participants had a history of antibiotic intake before their hospital visit, and all received inpatient antibiotic therapy based on the empirical assumption of bacterial meningitis. As bacterial meningitis is a rapidly fatal disease requiring immediate, aggressive intervention, this presumptive treatment approach creates substantial economic and clinical burdens. Specifically, managing non-bacterial or viral cases as bacterial infections leads to unnecessary hospital admissions and prolonged, costly intravenous broad-spectrum antibiotic courses. Consequently, this exposure increases patient vulnerability to severe drug side effects, antimicrobial resistance, institutional isolation, and profound indirect wage losses for both patients and family caregivers [5,32]. History of antibiotic intake before the hospital visit was associated with unfavorable outcomes (AOR = 2.784, 95% CI: 1.270–6.104). However, this association should be interpreted cautiously because of the potential for confounding by indication. Patients with more severe, prolonged, or atypical presentations may be more likely to receive antibiotics before hospital presentation and may independently be at greater risk of unfavorable outcomes.

Pre-hospital antibiotic use may also alter the clinical presentation and complicate subsequent clinical assessment and management. Therefore, the observed association should not be interpreted as evidence of a direct causal effect. Antibiotic therapy remains beneficial when appropriately indicated and targeted, underscoring the importance of appropriate antibiotic use and timely clinical assessment.

Given the likely presence of residual confounding in our analyses, these findings provide a foundation for further investigation into the roles of sex, delayed healthcare presentation, and prior antibiotic exposure in contributing to unfavorable outcomes in viral meningitis.

In our study, there were no significant differences in patient outcomes between those with identified viral etiology and those without.

Despite providing valuable insights, this study had limitations. We investigated only the most common viruses and cannot exclude the possibility that other rare, novel, or emerging viruses may have been responsible for some cases. Moreover, the use of qualitative conventional PCR rather than multiplex real-time PCR may have limited diagnostic sensitivity and increased the potential for contamination, and the uniform use of the standard GOS may have limited sensitivity for assessing outcomes in younger children. In addition, the predominantly pediatric study population may limit the generalizability of the findings to other age groups. The absence of follow-up beyond hospital discharge limits a comprehensive understanding of long-term patient outcomes.

## Conclusion

This predominantly pediatric study showed that EVs are the leading cause of viral meningitis in Ethiopia. While the majority of participants had favorable outcomes, unfavorable outcomes were significantly associated with antibiotic use before hospital presentation and delayed access to healthcare facilities. Based on our findings, we strongly recommend incorporating PCR into routine laboratory procedures to improve the detection and accurate identification of viral meningitis-causing pathogens, enabling earlier and improved management. Further identification and characterization of EVs are warranted.

## Supporting information

S1 Text Viral meningitis SPSS data. (SAV)

S2_row_images Original agarose gel electrophoresis image of PCR amplification product bands of enterovirus by PanEV2–5′NTR on a 2.5% agarose gel. From the left: Lane 1 = 100-bp DNA ladder; Lane 2 = positive control; Lanes 3 and 4 = enterovirus positive sample products 144 bp; and Lane 6 = NC, negative control respectively.
(PDF)

## References

[1] McGill F, Griffiths MJ, Solomon T. Viral meningitis: Current issues in diagnosis and treatment. Curr Opin Infect Dis. 2017;30(2):248–56. doi: 10.1097/QCO.0000000000000355 28118219

[2] Geteneh A, Kiros M, Tamrat E, Tesfaye A, Gashaw Y, Biset S, et al. Viral meningitis in Sub-Saharan Africa: Trends in prevalence, etiologies, and diagnostic approaches. Virol J. 2025;22(1):100. doi: 10.1186/s12985-025-02730-w 40234954 PMC12001642

[3] Kohil A, Jemmieh S, Smatti MK, Yassine HM. Viral meningitis: An overview. Arch Virol. 2021;166(2):335–45. doi: 10.1007/s00705-020-04891-1 33392820 PMC7779091

[4] Geteneh A, Kassa T, Alemu D, Kiros M, Andualem H, Tenna A, et al. Viral etiologies of meningitis in patients with presumed pyogenic meningitis at university hospitals in Ethiopia. Infect Drug Resist. 2021;14:1083–8. doi: 10.2147/IDR.S304521 PMC7982549 33762832

[5] Wami AA, Hundie GB, Ambachew R, Gebreyohannes Berhe Z, Abrha A, Abebe W, et al. High rate of human enteroviruses among clinically suspected meningitis cases at selected Hospitals in Addis Ababa, Ethiopia. PLoS One. 2021;16(11):e0258652. doi: 10.1371/journal.pone.0258652 34762656 PMC8584720

[6] Xu H, Chen P, Guo S, Shen X, Lu Y. Progress in etiological diagnosis of viral meningitis. Front Neurol. 2023;14:1193834. doi: 10.3389/fneur.2023.1193834 37583954 PMC10423822

[7] Ben Abid F, Abukhattab M, Ghazouani H, Khalil O, Gohar A, Al Soub H, et al. Epidemiology and clinical outcomes of viral central nervous system infections. Int J Infect Dis. 2018;73:85–90. doi: 10.1016/j.ijid.2018.06.008 29913285

[8] Ivaska L, Herberg J, Sadarangani M. Distinguishing community-acquired bacterial and viral meningitis: Microbes and biomarkers. J Infect. 2024;88(3):106111. doi: 10.1016/j.jinf.2024.01.010 38307149

[9] Petersen PT, Bodilsen J, Jepsen MPG, Larsen L, Storgaard M, Hansen BR, et al. Clinical features and prognostic factors in adults with viral meningitis. Brain. 2023;146(9):3816–25. doi: 10.1093/brain/awad089 36929167 PMC10473559

[10] Liu JJ, Teng LP, Hua CZ, Xie YP, Pan YX, Hu BF, et al. Etiological Analysis of Viral Encephalitis in Children in Zhejiang Province from 2018 to 2019. Diagnostics (Basel). 2022;12(8):1964. doi: 10.3390/diagnostics12081964 36010314 PMC9407060

[11] Pormohammad A, Goudarzi H, Eslami G, Falah F, Taheri F, Ghadiri N, et al. Epidemiology of herpes simplex and varicella zoster virus in cerebrospinal fluid of patients suffering from meningitis in Iran. New Microbes New Infect. 2020;36:100688. doi: 10.1016/j.nmni.2020.100688 32528686 PMC7276451

[12] Liu J, Yi Y, Chen W, Si S, Yin M, Jin H, et al. Development and evaluation of the quantitative real-time PCR assay in detection and typing of herpes simplex virus in swab specimens from patients with genital herpes. Int J Clin Exp Med. 2015;8(10):18758–64. 26770492 PMC4694392

[13] Wong AA, Pabbaraju K, Wong S, Tellier R. Development of a multiplex real-time PCR for the simultaneous detection of herpes simplex and varicella zoster viruses in cerebrospinal fluid and lesion swab specimens. J Virol Methods. 2016;229:16–23. doi: 10.1016/j.jviromet.2015.12.009 26711555

[14] McGill F, Griffiths MJ, Bonnett LJ, Geretti AM, Michael BD, Beeching NJ, et al. Incidence, aetiology, and sequelae of viral meningitis in UK adults: A multicentre prospective observational cohort study. Lancet Infect Dis. 2018;18(9):992–1003. doi: 10.1016/S1473-3099(18)30245-7 30153934 PMC6105576

[15] Wright WF, Pinto CN, Palisoc K, Baghli S. Viral (aseptic) meningitis: A review. J Neurol Sci. 2019;398:176–83. doi: 10.1016/j.jns.2019.01.050 30731305

[16] Moleleki M, Nel P, Matukane SR, Cloete S, Abrahams Z, Wolter N, et al. Enhanced pathogen identification among patients with clinically suspected meningitis. S Afr J Infect Dis. 2024;39(1):688. doi: 10.4102/sajid.v39i1.688 39822283 PMC11736564

[17] Salavatiha Z, Arefi A, Rabie M, Sobouti B, Nateghian A, Ataei-Pirkooh A, et al. The Prevalence of Enteroviruses, Mumps virus, and Herpes Simplex Virus-1 in Cerebrospinal Fluid Samples of Children with Aseptic Meningitis. Arch Razi Inst. 2024;79(5):1039–46. doi: 10.32592/ARI.2024.79.5.1039 40292054 PMC12018740

[18] de Oliveira DB, Candiani TM, Franco-Luiz APM, Almeida GMF, Abrahao JS, Rios M. Etiological agents of viral meningitis in children from a dengue-endemic area, Southeast region of Brazil. J Neurol Sci. 2017;375:390–4. doi: 10.1016/j.jns.2017.02.025 28320174

[19] Song P, Seok JM, Kim S, Choi J, Bae JY, Yu SN, et al. Viral etiology of aseptic meningitis and clinical prediction of herpes simplex virus type 2 meningitis. J Pers Med. 2024;14(9):998. doi: 10.3390/jpm14090998 39338252 PMC11433182

[20] Barnes GK, Gudina EK, Berhane M, Abdissa A, Tesfaw G, Abebe G, et al. New molecular tools for meningitis diagnostics in Ethiopia - A necessary step towards improving antimicrobial prescription. BMC Infect Dis. 2018;18(1):684. doi: 10.1186/s12879-018-3589-4 30572843 PMC6302510

[21] Farshadpour F, Taherkhani R. Molecular epidemiology of enteroviruses and predominance of echovirus 30 in an Iranian population with aseptic meningitis. J Neurovirol. 2021;27(3):444–51. doi: 10.1007/s13365-021-00973-1 33788142

[22] Jarrin I, Sellier P, Lopes A, Morgand M, Makovec T, Delcey V, et al. Etiologies and management of aseptic meningitis in patients admitted to an internal medicine department. Medicine (Baltimore). 2016;95(2):e2372. doi: 10.1097/MD.0000000000002372 26765411 PMC4718237

[23] Nahdi I, Boukoum H, Nabil Ben Salem A, Ben Romdane F, Hammami S, Chebel S, et al. Detection of herpes simplex virus (1 and 2), varicella-zoster virus, cytomegalovirus, human herpesvirus 6 and enterovirus in immunocompetent Tunisian patients with acute neuromeningeal disorder. J Med Virol. 2012;84(2):282–9. doi: 10.1002/jmv.23192 22170549

[24] Kim MG, Gulholm T, Lennard K, Mirdad F, Overton K, Maley M, et al. The impact of cerebrospinal fluid viral polymerase chain reaction testing on the management of adults with viral meningitis: A multi-center retrospective study. J Med Virol. 2023;95(1):e28198. doi: 10.1002/jmv.28198 36207770 PMC10092443

[25] Taherkhani R, Farshadpour F. Molecular identification and clinical characteristics of mumps virus and human herpesviruses associated with aseptic meningitis in South of Iran. Virusdisease. 2021;32(4):666–73. doi: 10.1007/s13337-021-00718-y 34901323 PMC8630270

[26] Adjei EF, Adiku TK, Mawuli G, Bonney JHK. Molecular investigations of viral meningitis among HIV-infected adults in Accra, Ghana. BMC Res Notes. 2018;11(1):615. doi: 10.1186/s13104-018-3720-z 30153867 PMC6114794

[27] Wang YL, Guo XT, Zhu MY, Mao YC, Xu XB, Hua Y, et al. Metagenomic next-generation sequencing and proteomics analysis in pediatric viral encephalitis and meningitis. Front Cell Infect Microbiol. 2023;13:1104858. doi: 10.3389/fcimb.2023.1104858 37153144 PMC10161730

[28] Aldriweesh MA, Shafaay EA, Alwatban SM, Alkethami OM, Aljuraisi FN, Bosaeed M, et al. Viruses causing aseptic meningitis: A tertiary medical center experience with a multiplex PCR assay. Front Neurol. 2020;11:602267. doi: 10.3389/fneur.2020.602267 33424752 PMC7793969

[29] Schwitter J, Branca M, Bicvic A, Abbuehl LS, Suter-Riniker F, Leib SL, et al. Long-term sequelae after viral meningitis and meningoencephalitis are frequent, even in mildly affected patients, a prospective observational study. Front Neurol. 2024;15:1411860. doi: 10.3389/fneur.2024.1411860 39087005 PMC11288970

[30] Bodilsen J, Mens H, Midgley S, Brandt CT, Petersen PT, Larsen L, et al. Enterovirus meningitis in adults: A prospective nationwide population-based cohort study. Neurology. 2021;97(5):e454–63. doi: 10.1212/WNL.0000000000012294 34088872

[31] Mesele DW, Kalayu AA, Teshome S, Aman J, Alemu A, Ayele L, et al. Enhanced detection of bacterial etiologies, antibiotic resistance profile, and treatment outcomes of meningitis patients in Ethiopia: A prospective cross-sectional study. BMC Microbiol. 2025;26(1):35. doi: 10.1186/s12866-025-04569-1 41387772 PMC12817866

[32] Chala TK, Lemma TD, Godana KT, Arefayine MB, Abdissa A, Gudina EK. The cost of suspected and confirmed bacterial meningitis cases treated at Jimma University Medical Center, Ethiopia. Ethiop J Health Sci. 2022;32(4):765–72. doi: 10.4314/ejhs.v32i4.13 PMC9341010 35950067

