# Supplementary material for: The hidden burden of viral meningitis in ethiopia: Etiology and clinical outcomes
Source: PLoS One. 2026 Oct 23;21(10):e0361291. doi: 10.1371/journal.pone.0361291 (PMC13693901; doi:10.1371/journal.pone.0361291)
Supplement: Supplementary file 1 [file pone.0361291.s002.pdf]

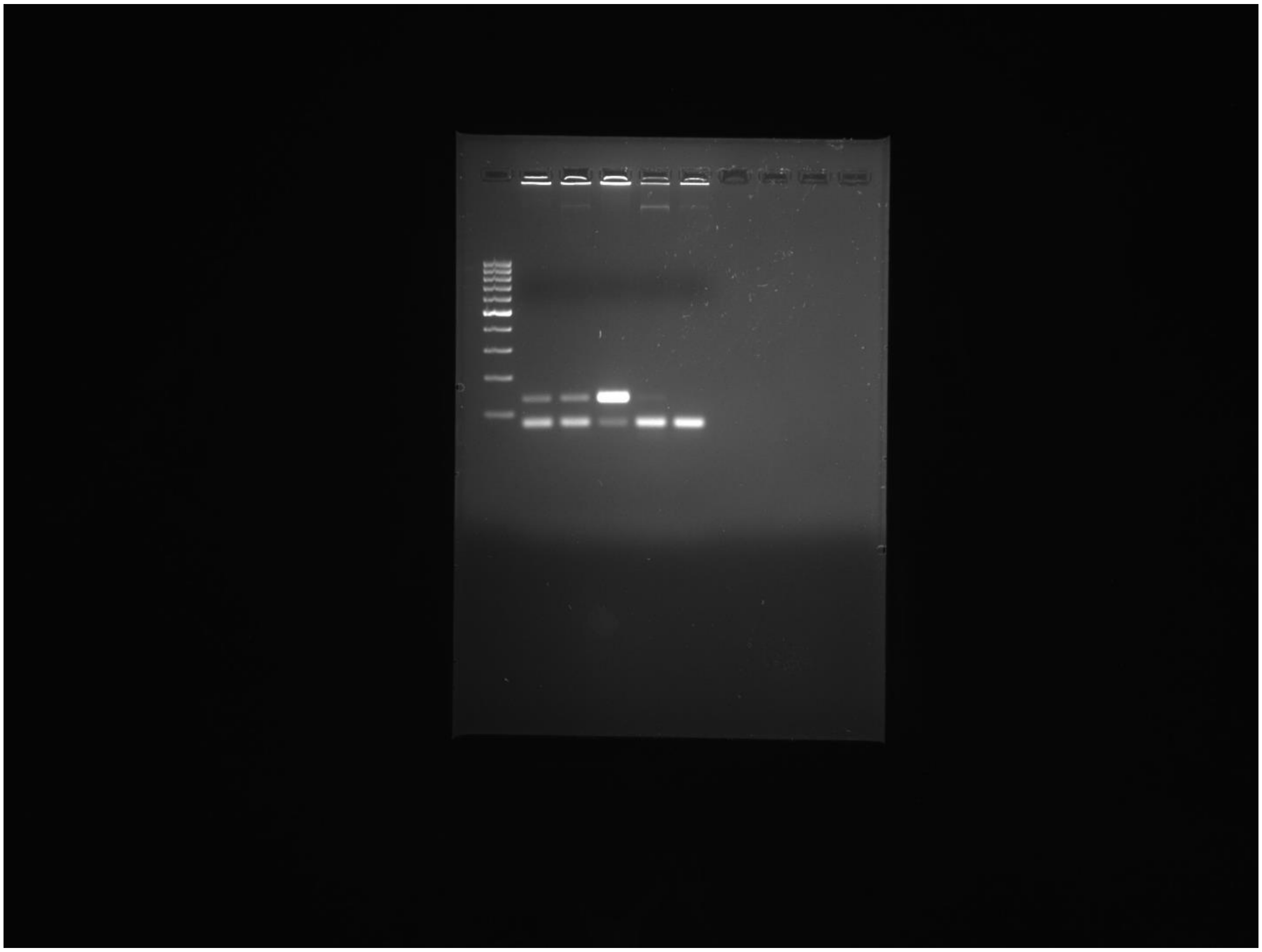

Original agarose gel electrophoresis image of PCR amplification product bands of enterovirus by PanEV2-5′NTR on a 2.5% agarose gel.

From the left: Lane 1 = 100-bp DNA ladder; Lane 2 = positive control; Lanes 3 and 4 = enterovirus-positive sample products 144 bp; and Lane 6 = NC, negative control respectively.
