# Supplementary material for: The hidden burden of viral meningitis in ethiopia: Etiology and clinical outcomes
Source: PLoS One. 2026 Oct 23;21(10):e0361291. doi: 10.1371/journal.pone.0361291 (PMC13693901; doi:10.1371/journal.pone.0361291)
Supplement: Supplementary file 3 [file pone.0361291.s005.docx]

**Response to Editors and Reviewers’ Comments**

**Dear Editors and Reviewers,**

We sincerely thank the editors and reviewers of PLOS ONE for their valuable time and constructive comments. Your insights have significantly improved the quality and clarity of our manuscript. We have carefully addressed all suggestions and revised the manuscript accordingly. Our point-by-point response follows, with all page and line numbers referring to the revised document. The changes we have made in the revised manuscript are highlighted in yellow.

**Reviewer 1**

| Editors Comment | Author Response |
| --- | --- |
| 1. 1. Please ensure that your manuscript meets PLOS ONE's style requirements, including those for file naming. | We sincerely thank the Academic Editor. We have ensured that all submitted files comply with PLOS ONE’s formatting and file-naming requirements, including the use of the following file names: “Response to Reviewers”, “Revised Manuscript with Track Changes”, and “Manuscript”. |
| 2. 2. We note that the grant information you provided in the ‘Funding Information’ and ‘Financial Disclosure’ sections do not match.   When you resubmit, please ensure that you provide the correct grant numbers for the awards you received for your study in the ‘Funding Information’ section. | Thank you. Now that we have reviewed the funding information and corrected the Financial Disclosure sections to ensure consistency and accuracy, including the correct grant numbers. |
| 3. 3. Thank you for stating the following financial disclosure:   “The study was supported by the European Union in collaboration with the African Academy of Sciences through the African Research Initiative for Scientific Excellence (ARISE) program and Addis Ababa University (AAU).” Please state what role the funders took in the study.  If the funders had no role, please state: "The funders had no role in study design, data collection and analysis, decision to publish, or preparation of the manuscript."  If this statement is not correct, you must amend it as needed.  Please include this amended Role of Funder statement in your cover letter; we will change the online submission form on your behalf. | We confirm that the funders had no role in the study design, data collection and analysis, decision to publish, or preparation of the manuscript. |
| 4. 4. Please provide a complete Data Availability Statement in the submission form, ensuring you include all necessary access information or a reason for why you are unable to make your data freely accessible. If your research concerns only data provided within your submission, please write "All data are in the manuscript and/or supporting information files" as your Data Availability Statement. | We confirm that the data availability statement has been revised to clarify that the data supporting the findings of this study are available from the corresponding author upon reasonable request. |
| 5. 5. If the reviewer comments include a recommendation to cite specific previously published works, please review and evaluate these publications to determine whether they are relevant and should be cited. There is no requirement to cite these works unless the editor has indicated otherwise. | We note that there were no specific recommendations for additional citations. If such recommendations are provided, we will carefully assess their relevance and cite them only where appropriate and necessary. |
| Additional Editor Comments  There were no indications as you performed  nucleic acid amplification technique to identify viral causes of meningitis, for example, gel documentation for the identified viral case of meningitis. | We confirm that nucleic acid amplification testing was performed for the identification of viral causes of meningitis. If required, we would be pleased to provide the gel electrophoresis images of the PCR results for the identified viral cases. |

**Reviewer 1**

| This is a commendable and necessary multicenter study detailing the viral etiologies of meningitis in Ethiopia. The data provides a solid foundation for refining local empirical treatment guidelines. However, several aspects of the methodology and data interpretation require critical revision to ensure clinical precision. | | |
| --- | --- | --- |
| Reviewer Comment | Author Response | Revision / Location |
| Major Comments   1. Cohort Demographics: The manuscript data indicates that 90.5% of the study participants are children under 18 years of age, with 74% being 5 years old or younger. The title, abstract, and introduction must be revised to accurately reflect that this is overwhelmingly a pediatric cohort. Presenting the findings as representative of the general population without this qualification is epidemiologically imprecise. | We sincerely thank you for this important observation. We have revised the Abstract, Introduction, and Conclusion to clearly reflect the predominantly pediatric composition of the study population. We have also explicitly acknowledged this consideration in the Limitations section and clarified that the findings should be interpreted accordingly. | Page 2, Lines 45  Page 4, Lines 73 & 89  Page 16, Lines 320  Page 16, Lines 324 |
| 2. Outcome Measurement Validity: Patient outcomes were evaluated using the standard Glasgow Outcome Scale (GOS). Given that the vast majority of your patients are infants and toddlers, the standard adult GOS lacks sensitivity and developmental appropriateness. Please clarify in the Methods section if a pediatric-validated version of the scale was utilized, and if not, this must be stated as a prominent limitation. | Thank you very much for this important observation. We confirm that the standard Glasgow Outcome Scale (GOS) was used uniformly across the entire cohort; a pediatric-specific validated version was not used. We deliberately applied a single standardized outcome measure to ensure consistency and comparability across participants of different ages. However, we acknowledge that the standard GOS may lack sensitivity to developmental differences and age-specific functional outcomes in young children. This limitation has now been explicitly acknowledged in the Limitations section of the revised manuscript. | Page 16, Lines 319 & 320 |
| 3. Confounding Variables: The association between a history of antibiotic intake before the hospital visit and unfavorable outcomes (AOR = 2.784) is framed as a direct risk factor. From an evidence-based perspective, this analysis is highly susceptible to confounding by indication. Patients presenting with more severe, prolonged, or atypical initial symptoms are inherently more likely to have sought prior interventions (antibiotics) and concurrently more likely to experience unfavorable outcomes. The Discussion section must explicitly address this confounding reality rather than heavily implying a strictly causal relationship. | We agree that the association between pre-hospital antibiotic use and unfavorable outcomes may be subject to confounding by indication. Patients with more severe, prolonged, or atypical clinical presentations may be more likely to receive antibiotics before hospital presentation, and independently, may have a greater likelihood of unfavorable outcomes. Thus, the observed association (AOR = 2.784, 95% CI: 1.270–6.104) should not be interpreted as evidence of a direct causal effect of pre-hospital antibiotic use on clinical outcomes.  Accordingly, we have revised the Discussion to explicitly acknowledge the potential for confounding by indication and have moderated the causal interpretation of this finding. We now describe pre-hospital antibiotic use as an associated factor rather than a direct risk factor and acknowledge that the observed relationship may partly reflect differences in underlying disease severity and clinical presentation. | Page 15 &16, Lines 300-309 |
| 4. Diagnostic Limitations: The reliance on qualitative conventional polymerase chain reaction (PCR) rather than multiplex Real-Time PCR (qPCR) introduces known risks of lower sensitivity and potential cross-contamination. While acceptable for resource-limited settings, this technical limitation must be formally acknowledged in the Discussion section. | We sincerely thank you for highlighting this diagnostic limitation. We agree that the use of qualitative conventional PCR, rather than multiplex real-time PCR (qPCR), may have limitations in terms of diagnostic sensitivity and contamination control. Given the resource-limited setting in which the study was conducted, conventional PCR was used as the available molecular diagnostic approach. We have now explicitly acknowledged this limitation in the Discussion and noted that the findings should be interpreted in light of the diagnostic method we used. | Page 16, Lines 317-319 |
| Minor Comments Ensure that all reporting of odds ratios, confidence intervals, and p-values in the main text precisely matches the data presented in Table 5. The absence of Varicella-Zoster Virus (VZV) and Mumps virus detections is notable. Briefly expand on the specific implications of missing atypical or emerging viral agents due to the targeted limits of the chosen primer panel. | We carefully cross-checked all odds ratios, 95% confidence intervals, and *p*-values reported in the main text with Table 5 and corrected any discrepancies to ensure complete consistency.  We acknowledge that the targeted primer panel was limited to the selected viral pathogens and therefore may not have detected atypical, rare, or emerging viral agents. We have clarified this limitation in the revised Discussion and noted that undetected viral etiologies cannot be excluded. | Page 14, Lines 265-269  Page 16, Lines 315-317 |

**Reviewer 2**

| Reviewer Comment | Author Response | Revision / Location |
| --- | --- | --- |
| This is a well-designed multicenter study assessing viral meningitis etiologies and patient outcomes in Ethiopia. The methodology is solid, and the findings are clinically relevant. I recommend Minor Revision to address the following minor points: Pediatric Dominance: Around 90.5% of the study population are children (<18 years). Please emphasize this demographic distribution in the Discussion, as it heavily influences the etiology (e.g., high enterovirus prevalence) and outcomes. Unfavorable Outcomes & Sample Size: Since only 29 patients experienced unfavorable outcomes (GOS 1–4), multivariate analysis for associated factors might be slightly constrained. Please mention this potential limitation in the Discussion. Typographical & Formatting Checks: Minor typos should be revised throughout the text (e.g., "ditected" in the Table 4 header, and alignment in the abstract/methods). | We sincerely thank the reviewer for the positive assessment of our study and for the constructive feedback and suggestions. We have addressed all of the comments as follows:  **1. Pediatric Dominance:** We have emphasized the predominantly pediatric composition of the study population in the Discussion and acknowledged that this demographic distribution should be considered when interpreting the observed viral etiology and clinical outcomes.  **2. Unfavorable Outcomes and Sample Size:** We agree that the relatively small number of patients with unfavorable outcomes (n = 29) may have limited the precision and statistical power of the multivariable analysis. This limitation has been explicitly acknowledged in the revised Discussion.  **3. Typographical and Formatting Checks:** We have carefully reviewed the manuscript and corrected the identified typographical and formatting errors, including the typo “ditected” in the Table 4 header and inconsistencies in alignment within the Abstract and Methods sections. | Page 2, Lines 45  Page 4, Lines 73  Page 16, Lines 320  Page 11, Lines 209 & 214 |

Sincerely,

Derso Wale Mesele

On behalf of all authors
