# Supplementary material for: The hidden burden of viral meningitis in ethiopia: Etiology and clinical outcomes
Source: PLoS One. 2026 Oct 23;21(10):e0361291. doi: 10.1371/journal.pone.0361291 (PMC13693901; doi:10.1371/journal.pone.0361291)
Supplement: Supplementary file 4 [file pone.0361291.s004.docx]

Among the comments given the following three have been modified and/or explained well in the revised edition. I hope justification for the other feed backs have reached the editor in the point by point response.

1^st^ is the correction with the number numerator and denominator regarding prevalence of viral meningitis in line 203.

2^nd^ is justification as to how prior antibiotics intake could be risk for complication is explained in line 299-304.

3^rd^ is conclusion against recommending empiric antibiotics use is also corrected which was stated in line 317.

The rest are not well explained.
