# Supplementary material for: The hidden burden of viral meningitis in ethiopia: Etiology and clinical outcomes
Source: PLoS One. 2026 Oct 23;21(10):e0361291. doi: 10.1371/journal.pone.0361291 (PMC13693901; doi:10.1371/journal.pone.0361291)
Supplement: Supplementary file 5 [file pone.0361291.s006.docx]

**Dear Editor**

Thank you for your feedback and constructive comments on our manuscript. We have carefully addressed the reviewers' suggestions to improve the quality and clarity of our paper. We attached our Revised Manuscript with Track Changes—with all changes highlighted in yellow—along with a point-by-point response citing the exact page and line numbers.

Reviewers 1, 2, and 3 are in agreement with our revised manuscript. However, regarding the decision letter sent on August 12, 2026, we received the detailed comments from Reviewers 1 and 2, but the feedback from Reviewers 3 and 4 was not included in the correspondence.

In the current version, we were able to review and address part of Reviewer 4's concerns based on their summary response. However, to ensure we fully address all feedback, could you kindly resend the complete review comments for Reviewer 4, or advise us on how you would like us to proceed? Looking forward to hearing your decision.

Thank you again for your time, support, and guidance.

**Additional Editor Comments**

If you have gel documentation at hand, provide it as a figure in a separate file, prepared as a TIF/TIFF version; here, the caption would not be in the figure. The figure should be cited in the manuscript, and the caption is also placed within it.

**Response**

We sincerely thank you for your suggestions. We have provided the gel electrophoresis documentation as a separate TIF figure file. The figure is cited in the manuscript (page 10, line 207), and the corresponding caption is provided separately in the manuscript (page 11, lines 210 – 212).

Sincerely,

Derso Wale Mesele

On behalf of all authors
