# Supplementary material for: The hidden burden of viral meningitis in ethiopia: Etiology and clinical outcomes
Source: PLoS One. 2026 Oct 23;21(10):e0361291. doi: 10.1371/journal.pone.0361291 (PMC13693901; doi:10.1371/journal.pone.0361291)
Supplement: Peer review history

# Peer review: Decision Letter 0

**DOI:** 10.1371/journal.pone.0361291.r001

**Authors:** Tebelay Dilnessa

11 Aug 2026

Dear Dr. Mesele,

Thank you for submitting your manuscript to PLOS ONE. After careful consideration, we feel that it has merit but does not fully meet PLOS ONE’s publication criteria as it currently stands. Therefore, we invite you to submit a revised version of the manuscript that addresses the points raised during the review process.

Please submit your revised manuscript by Oct 10 2026 11:59PM. If you will need more time than this to complete your revisions, please reply to this message or contact the journal office at plosone@plos.org. When you're ready to submit your revision, log on to https://www.editorialmanager.com/pone/ and select the 'Submissions Needing Revision' folder to locate your manuscript file.

- A letter that responds to each point raised by the academic editor and reviewer(s). You should upload this letter as a separate file labeled 'Response to Reviewers'.
- A marked-up copy of your manuscript that highlights changes made to the original version. You should upload this as a separate file labeled 'Revised Manuscript with Track Changes'.
- An unmarked version of your revised paper without tracked changes. You should upload this as a separate file labeled 'Manuscript'.

If you would like to make changes to your financial disclosure, please include your updated statement in your cover letter. Guidelines for resubmitting your figure files are available below the reviewer comments at the end of this letter.

If applicable, we recommend that you deposit your laboratory protocols in protocols.io to enhance the reproducibility of your results. Protocols.io assigns your protocol its own identifier (DOI) so that it can be cited independently in the future. For instructions see: https://journals.plos.org/plosone/s/submission-guidelines#loc-laboratory-protocols. Additionally, PLOS ONE offers an option for publishing peer-reviewed Lab Protocol articles, which describe protocols hosted on protocols.io. Read more information on sharing protocols at https://plos.org/protocols?utm_medium=editorial-email&utm_source=authorletters&utm_campaign=protocols.

As the corresponding author, your ORCID iD is verified in the submission system and will appear in the published article. PLOS supports the use of ORCID, and we encourage all coauthors to register for an ORCID iD and use it as well. Please encourage your coauthors to verify their ORCID iD within the submission system before final acceptance, as unverified ORCID iDs will not appear in the published article. *Only*  the individual author can complete the verification step; PLOS staff *cannot*  verify ORCID iDs on behalf of authors.

We look forward to receiving your revised manuscript.

Kind regards,

Tebelay Dilnessa, MSc

Academic Editor

PLOS One

Journal Requirements:

When submitting your revision, we need you to address these additional requirements.

1. Please ensure that your manuscript meets PLOS ONE's style requirements, including those for file naming. The PLOS ONE style templates can be found at

https://journals.plos.org/plosone/s/file?id=wjVg/PLOSOne_formatting_sample_main_body.pdf and

https://journals.plos.org/plosone/s/file?id=ba62/PLOSOne_formatting_sample_title_authors_affiliations.pdf

2. We note that the grant information you provided in the ‘Funding Information’ and ‘Financial Disclosure’ sections do not match.

When you resubmit, please ensure that you provide the correct grant numbers for the awards you received for your study in the ‘Funding Information’ section.

3. Thank you for stating the following financial disclosure:

“The study was supported by the European Union in collaboration with the African Academy of Sciences through the African Research Initiative for Scientific Excellence (ARISE) program and Addis Ababa University (AAU).”

Please state what role the funders took in the study.  If the funders had no role, please state: "The funders had no role in study design, data collection and analysis, decision to publish, or preparation of the manuscript."

If this statement is not correct you must amend it as needed.

Please include this amended Role of Funder statement in your cover letter; we will change the online submission form on your behalf.

4. Please provide a complete Data Availability Statement in the submission form, ensuring you include all necessary access information or a reason for why you are unable to make your data freely accessible. If your research concerns only data provided within your submission, please write "All data are in the manuscript and/or supporting information files" as your Data Availability Statement.

5. If the reviewer comments include a recommendation to cite specific previously published works, please review and evaluate these publications to determine whether they are relevant and should be cited. There is no requirement to cite these works unless the editor has indicated otherwise.

Additional Editor Comments:

- There were no indications as you performed nucleic acid amplification technique to identify viral causes of meningitis, for example, gel documentation for the identified viral case of  meningitis.

[Note: HTML markup is below. Please do not edit.]

Reviewers' comments:

Reviewer's Responses to Questions

**Comments to the Author**

1. Is the manuscript technically sound, and do the data support the conclusions?

Reviewer #1: Partly

Reviewer #2: Yes

**********

2. Has the statistical analysis been performed appropriately and rigorously?

Reviewer #1: No

Reviewer #2: Yes

**********

3. Have the authors made all data underlying the findings in their manuscript fully available?

The PLOS Data policy

Reviewer #1: Yes

Reviewer #2: Yes

**********

4. Is the manuscript presented in an intelligible fashion and written in standard English?

Reviewer #1: Yes

Reviewer #2: Yes

**********

Reviewer #1: Comments to the Authors

This is a commendable and necessary multicenter study detailing the viral etiologies of meningitis in Ethiopia. The data provides a solid foundation for refining local empirical treatment guidelines. However, several aspects of the methodology and data interpretation require critical revision to ensure clinical precision.

Major Comments

1. Cohort Demographics: The manuscript data indicates that 90.5% of the study participants are children under 18 years of age, with 74% being 5 years old or younger. The title, abstract, and introduction must be revised to accurately reflect that this is overwhelmingly a pediatric cohort. Presenting the findings as representative of the general population without this qualification is epidemiologically imprecise.

2. Outcome Measurement Validity: Patient outcomes were evaluated using the standard Glasgow Outcome Scale (GOS). Given that the vast majority of your patients are infants and toddlers, the standard adult GOS lacks sensitivity and developmental appropriateness. Please clarify in the Methods section if a pediatric-validated version of the scale was utilized, and if not, this must be stated as a prominent limitation.

3. Confounding Variables: The association between a history of antibiotic intake before the hospital visit and unfavorable outcomes (AOR = 2.784) is framed as a direct risk factor. From an evidence-based perspective, this analysis is highly susceptible to confounding by indication. Patients presenting with more severe, prolonged, or atypical initial symptoms are inherently more likely to have sought prior interventions (antibiotics) and concurrently more likely to experience unfavorable outcomes. The Discussion section must explicitly address this confounding reality rather than heavily implying a strictly causal relationship.

4. Diagnostic Limitations: The reliance on qualitative conventional polymerase chain reaction (PCR) rather than multiplex Real-Time PCR (qPCR) introduces known risks of lower sensitivity and potential cross-contamination. While acceptable for resource-limited settings, this technical limitation must be formally acknowledged in the Discussion section.

Minor Comments

Ensure that all reporting of odds ratios, confidence intervals, and p-values in the main text precisely matches the data presented in Table 5.

The absence of Varicella-Zoster Virus (VZV) and Mumps virus detections is notable. Briefly expand on the specific implications of missing atypical or emerging viral agents due to the targeted limits of the chosen primer panel.

Reviewer #2: This is a well-designed multicenter study assessing viral meningitis etiologies and patient outcomes in Ethiopia. The methodology is solid, and the findings are clinically relevant. I recommend Minor Revision to address the following minor points: Pediatric Dominance: Around 90.5% of the study population are children (<18 years). Please emphasize this demographic distribution in the Discussion, as it heavily influences the etiology (e.g., high enterovirus prevalence) and outcomes. Unfavorable Outcomes & Sample Size: Since only 29 patients experienced unfavorable outcomes (GOS 1–4), multivariate analysis for associated factors might be slightly constrained. Please mention this potential limitation in the Discussion. Typographical & Formatting Checks: Minor typos should be revised throughout the text (e.g., "ditected" in the Table 4 header, and alignment in the abstract/methods).

**********

what does this mean?). If published, this will include your full peer review and any attached files.

If you choose “no”, your identity will remain anonymous but your review may still be made public.

**Do you want your identity to be public for this peer review?**  For information about this choice, including consent withdrawal, please see our Privacy Policy

Reviewer #1: **Yes:**  Dr. Ahmadreza Ahmadi

Reviewer #2: No

**********

[NOTE: If reviewer comments were submitted as an attachment file, they will be attached to this email and accessible via the submission site. Please log into your account, locate the manuscript record, and check for the action link "View Attachments". If this link does not appear, there are no attachment files.]

To ensure your figures meet our technical requirements, please review our figure guidelines: https://journals.plos.org/plosone/s/figures

You may also use PLOS’s free figure tool, NAAS, to help you prepare publication quality figures: https://journals.plos.org/plosone/s/figures#loc-tools-for-figure-preparation.

NAAS will assess whether your figures meet our technical requirements by comparing each figure against our figure specifications.

# Author comment: Author response to Decision Letter 1

**DOI:** 10.1371/journal.pone.0361291.r002

29 Aug 2026

No specific comment.

Attachment Submitted filename: Response to Reviewers.docx

# Peer review: Decision Letter 1

**DOI:** 10.1371/journal.pone.0361291.r003

**Authors:** Tebelay Dilnessa

24 Sep 2026

Dear Dr. Mesele,

Thank you for submitting your manuscript to PLOS One. After careful consideration, we feel that it has merit but does not fully meet PLOS One’s publication criteria as it currently stands. Therefore, we invite you to submit a revised version of the manuscript that addresses the points raised during the review process.

Please submit your revised manuscript by Oct 24 2026 11:59PM. If you will need more time than this to complete your revisions, please reply to this message or contact the journal office at plosone@plos.org. When you're ready to submit your revision, log on to https://www.editorialmanager.com/pone/ and select the 'Submissions Needing Revision' folder to locate your manuscript file.

- A letter that responds to each point raised by the academic editor and reviewer(s). You should upload this letter as a separate file labeled 'Response to Reviewers'.
- A marked-up copy of your manuscript that highlights changes made to the original version. You should upload this as a separate file labeled 'Revised Manuscript with Track Changes'.
- An unmarked version of your revised paper without tracked changes. You should upload this as a separate file labeled 'Manuscript'.

If you would like to make changes to your financial disclosure, please include your updated statement in your cover letter. Guidelines for resubmitting your figure files are available below the reviewer comments at the end of this letter.

If applicable, we recommend that you deposit your laboratory protocols in protocols.io to enhance the reproducibility of your results. Protocols.io assigns your protocol its own identifier (DOI) so that it can be cited independently in the future. For instructions see: https://journals.plos.org/plosone/s/submission-guidelines#loc-laboratory-protocols. Additionally, PLOS One offers an option for publishing peer-reviewed Lab Protocol articles, which describe protocols hosted on protocols.io. Read more information on sharing protocols at https://plos.org/protocols?utm_medium=editorial-email&utm_source=authorletters&utm_campaign=protocols.

As the corresponding author, your ORCID iD is verified in the submission system and will appear in the published article. PLOS supports the use of ORCID, and we encourage all coauthors to register for an ORCID iD and use it as well. Please encourage your coauthors to verify their ORCID iD within the submission system before final acceptance, as unverified ORCID iDs will not appear in the published article. *Only*  the individual author can complete the verification step; PLOS staff *cannot*  verify ORCID iDs on behalf of authors.

We look forward to receiving your revised manuscript.

Kind regards,

Tebelay Dilnessa, MSc

Academic Editor

PLOS One

**Journal Requirements:**

1. If the reviewer comments include a recommendation to cite specific previously published works, please review and evaluate these publications to determine whether they are relevant and should be cited. There is no requirement to cite these works unless the editor has indicated otherwise.

2. Please review your reference list to ensure that it is complete and correct. If you have cited papers that have been retracted, please include the rationale for doing so in the manuscript text, or remove these references and replace them with relevant current references. Any changes to the reference list should be mentioned in the rebuttal letter that accompanies your revised manuscript. If you need to cite a retracted article, indicate the article’s retracted status in the References list and also include a citation and full reference for the retraction notice.

**Additional Editor Comments:**

- If you have gel documentation at hand, provide it as a figure in a separate file, prepared as a TIF/TIFF version; here, the caption would not be in the figure. The figure should be cited in the manuscript, and the caption is also placed within it.

[Note: HTML markup is below. Please do not edit.]

Reviewers' comments:

Reviewer's Responses to Questions

**Comments to the Author**

Reviewer #1: All comments have been addressed

Reviewer #3: (No Response)

Reviewer #4: All comments have been addressed

**********

2. Is the manuscript technically sound, and do the data support the conclusions?

Reviewer #1: Yes

Reviewer #3: Yes

Reviewer #4: Yes

**********

3. Has the statistical analysis been performed appropriately and rigorously?

Reviewer #1: Yes

Reviewer #3: Yes

Reviewer #4: Yes

**********

4. Have the authors made all data underlying the findings in their manuscript fully available?

The PLOS Data policy

Reviewer #1: Yes

Reviewer #3: No

Reviewer #4: (No Response)

**********

5. Is the manuscript presented in an intelligible fashion and written in standard English?

Reviewer #1: Yes

Reviewer #3: Yes

Reviewer #4: (No Response)

**********

Reviewer #1: Letter to the Authors

The modifications in your revised manuscript successfully address the methodological limitations and interpretative boundaries required for clinical precision.

Explicitly framing the cohort as overwhelmingly pediatric and acknowledging the developmental sensitivity limits of the standard GOS provides necessary context for your outcome data.

Your revision regarding the association between pre-hospital antibiotic use and unfavorable outcomes (AOR=2.784) is a critical correction. By acknowledging confounding by indication—specifically that patients with more severe or atypical initial presentations inherently seek prior interventions—you prevent the misinterpretation of this association as strictly causal.

Finally, formally noting the diagnostic limitations of qualitative conventional PCR, including potential sensitivity and cross-contamination risks, ensures the findings are interpreted appropriately within the context of a resource-limited setting. The manuscript is now methodologically sound.

Reviewer #3: Thank you for the invitation to review the manuscript titled "The Hidden Burden of Viral Meningitis in Ethiopia: Etiology and Clinical Outcomes." I have examined the revised version and note that the authors have satisfactorily addressed all previous comments. The manuscript is now well-prepared, and I recommend its approval for publication.

Reviewer #4: Among the comments given the following three have been modified and/or explained well in the revised edition. I hope justification for the other feed backs have reached the editor in the point by point response.

1st is the correction with the number numerator and denominator regarding prevalence of viral meningitis in line 203.

2nd is justification as to how prior antibiotics intake could be risk for complication is explained in line 299-304.

3rd is conclusion against recommending empiric antibiotics use is also corrected which was stated in line 317.

The rest are not well explained.

**********

what does this mean?). If published, this will include your full peer review and any attached files.

If you choose “no”, your identity will remain anonymous but your review may still be made public.

**Do you want your identity to be public for this peer review?**  For information about this choice, including consent withdrawal, please see our Privacy Policy

Reviewer #1: **Yes:**  Dr. Ahmadreza Ahmadi

Reviewer #3: No

Reviewer #4: No

**********

[NOTE: If reviewer comments were submitted as an attachment file, they will be attached to this email and accessible via the submission site. Please log into your account, locate the manuscript record, and check for the action link "View Attachments". If this link does not appear, there are no attachment files.]

To ensure your figures meet our technical requirements, please review our figure guidelines: https://journals.plos.org/plosone/s/figures

You may also use PLOS’s free figure tool, NAAS, to help you prepare publication quality figures: https://journals.plos.org/plosone/s/figures#loc-tools-for-figure-preparation.

NAAS will assess whether your figures meet our technical requirements by comparing each figure against our figure specifications.

Attachment Submitted filename: viral meningitis revised document comment.docx

# Author comment: Author response to Decision Letter 2

**DOI:** 10.1371/journal.pone.0361291.r004

7 Oct 2026

Dear Editor

Thank you for your feedback and constructive comments on our manuscript. We have carefully addressed the reviewers' suggestions to improve the quality and clarity of our paper. We attached our Revised Manuscript with Track Changes—with all changes highlighted in yellow—along with a point-by-point response citing the exact page and line numbers.

Reviewers 1, 2, and 3 are in agreement with our revised manuscript. However, regarding the decision letter sent on August 12, 2026, we received the detailed comments from Reviewers 1 and 2, but the feedback from Reviewers 3 and 4 was not included in the correspondence.

In the current version, we were able to review and address part of Reviewer 4's concerns based on their summary response. However, to ensure we fully address all feedback, could you kindly resend the complete review comments for Reviewer 4, or advise us on how you would like us to proceed? Looking forward to hearing your decision.

Thank you again for your time, support, and guidance.

Additional Editor Comments

If you have gel documentation at hand, provide it as a figure in a separate file, prepared as a TIF/TIFF version; here, the caption would not be in the figure. The figure should be cited in the manuscript, and the caption is also placed within it.

Response

We sincerely thank you for your suggestions. We have provided the gel electrophoresis documentation as a separate TIF figure file. The figure is cited in the manuscript (page 10, line 207), and the corresponding caption is provided separately in the manuscript (page 11, lines 210 – 212).

Attachment Submitted filename: Response_to_Reviewers_auresp_2.docx

# Editor report: Decision Letter 2

**DOI:** 10.1371/journal.pone.0361291.r005

**Authors:** Tebelay Dilnessa

9 Oct 2026

The Hidden Burden of Viral Meningitis in Ethiopia: Etiology and Clinical Outcomes.

PONE-D-26-34191R2

Dear Dr. Mesele,

We’re pleased to inform you that your manuscript has been judged scientifically suitable for publication and will be formally accepted for publication once it meets all outstanding technical requirements.

Within one week, you’ll receive an e-mail detailing the required amendments. When these have been addressed, you’ll receive a formal acceptance letter and your manuscript will be scheduled for publication.

An invoice will be generated when your article is formally accepted. Please note, if your institution has a publishing partnership with PLOS and your article meets the relevant criteria, all or part of your publication costs will be covered. Please make sure your user information is up-to-date by logging into Editorial Manager at Editorial Manager® and clicking the ‘Update My Information' link at the top of the page. For questions related to billing, please contact billing support.

If your institution or institutions have a press office, please notify them about your upcoming paper to help maximize its impact. If they’ll be preparing press materials, please inform our press team as soon as possible -- no later than 48 hours after receiving the formal acceptance. Your manuscript will remain under strict press embargo until 2 pm Eastern Time on the date of publication. For more information, please contact onepress@plos.org.

Kind regards,

Tebelay Dilnessa, MSc

Academic Editor

PLOS One

Additional Editor Comments (optional):

Reviewers' comments:

# Editor report: Acceptance letter

**DOI:** 10.1371/journal.pone.0361291.r006

**Authors:** Tebelay Dilnessa

PONE-D-26-34191R2

PLOS One

Dear Dr. Mesele,

I'm pleased to inform you that your manuscript has been deemed suitable for publication in PLOS One. Congratulations! Your manuscript is now being handed over to our production team.

At this stage, our production department will prepare your paper for publication. This includes ensuring the following:

* All references, tables, and figures are properly cited

* All relevant supporting information is included in the manuscript submission,

* There are no issues that prevent the paper from being properly typeset

You will receive further instructions from the production team, including instructions on how to review your proof when it is ready. Please keep in mind that we are working through a large volume of accepted articles, so please give us a few days to review your paper and let you know the next and final steps.

Lastly, if your institution or institutions have a press office, please let them know about your upcoming paper now to help maximize its impact. If they'll be preparing press materials, please inform our press team within the next 48 hours. Your manuscript will remain under strict press embargo until 2 pm Eastern Time on the date of publication. For more information, please contact onepress@plos.org.

You will receive an invoice from PLOS for your publication fee after your manuscript has reached the completed accept phase. If you receive an email requesting payment before acceptance or for any other service, this may be a phishing scheme. Learn how to identify phishing emails and protect your accounts at https://explore.plos.org/phishing.

If we can help with anything else, please email us at customercare@plos.org.

Thank you for submitting your work to PLOS One and supporting open access.

Kind regards,

PLOS One Editorial Office Staff

on behalf of

Dr. Tebelay Dilnessa

Academic Editor

PLOS One
